# High Negative Predictive Value but Moderate Overall Performance of Synovasure Compared With Joint Aspirate Culture in the Diagnosis of Prosthetic Joint Infection

**DOI:** 10.7759/cureus.101318

**Published:** 2026-01-11

**Authors:** Miles W Benjamin, Muddasir Reyaz Hassan, Abdirahman Osman, Nikolaos Papadakos, Aine Ringrose, John Stammers, Philip Mitchell, Sulaiman Alazzawi, Suroosh Madanipour

**Affiliations:** 1 Trauma and Orthopaedics, Epsom and St Helier University Hospitals NHS Trust, London, GBR; 2 Trauma and Orthopaedics, St George’s University Hospitals NHS Foundation Trust, London, GBR; 3 General Practice, North East London NHS Foundation Trust, London, GBR; 4 Radiology, St George’s University Hospitals NHS Foundation Trust, London, GBR; 5 Trauma and Orthopaedics, Royal National Orthopaedic Hospital, london, GBR; 6 Orthopaedics, St George’s University Hospitals NHS Foundation Trust, London, GBR

**Keywords:** alpha-defensin lateral flow, joint aspirate culture, peri-prosthetic joint infection, prosthetic hip joint infection, prosthetic joint infection (pji), prosthetic knee joint infection, synovasure

## Abstract

Accurate diagnosis of periprosthetic joint infection (PJI) is crucial for optimising treatment outcomes and reducing patient morbidity, mortality, and healthcare costs. This retrospective single-centre study assessed the diagnostic performance of the Synovasure alpha-defensin lateral flow (ADLF) test compared with conventional microbiological culture, which served as the reference standard. Between April 2016 and January 2019, 143 joints with suspected PJI underwent aspiration and Synovasure testing, with intraoperative tissue culture data included where available. The Synovasure ADLF test demonstrated a sensitivity of 78.95% and a specificity of 72.38%, indicating moderate diagnostic accuracy. Importantly, it achieved a high negative predictive value (90.48%), supporting its reliability in excluding infection, whereas the positive predictive value was lower (50.85%), limiting its utility as a confirmatory test when used in isolation. These findings suggest that while Synovasure provides rapid results and can be a valuable adjunct for ruling out infection, it should not replace established diagnostic methods. The test’s moderate sensitivity and specificity underline the need for cautious interpretation in clinical practice. Integrating Synovasure results with other diagnostic modalities, such as microbiological cultures, histopathology, and clinical assessment, remains essential for achieving accurate diagnosis and guiding appropriate management of PJI.

## Introduction and background

Periprosthetic joint infection (PJI) is one of the most serious complications following joint arthroplasty, resulting in significant functional impairment, prolonged hospital stays, and increased healthcare costs. In the United Kingdom, over 200,000 primary hip and knee arthroplasties were performed in 2018 alone, and the burden of revision procedures due to PJI continues to rise annually [1]. Reported infection rates following joint arthroplasty range between 0.7% and 2.4% [2]. These infections not only cause substantial morbidity and, in severe cases, mortality, but also carry a significant economic impact, with treatment costs frequently exceeding £18,000 per case [3].

The diagnosis of PJI remains complex due to the heterogeneity of its clinical presentation and the limitations of currently available diagnostic modalities. Joint aspiration with synovial fluid analysis and microbiological culture has long been considered the standard initial investigation. However, the diagnostic accuracy of culture can be highly variable, with reported sensitivity ranging from 12% to 89% and specificity from 50% to 100% [4,5]. Factors contributing to variability include previous antibiotic administration, which can suppress bacterial growth, and contamination during the aspiration procedure [6].

Alpha-defensin, a peptide released by neutrophils in response to bacterial infection, was first published in 2013 by Bingham et al. as a promising biomarker for PJI diagnosis. Synovasure (Zimmer Biomet, Warsaw, IN), a commercially available lateral flow assay, allows rapid detection of alpha-defensin in synovial fluid, providing results within minutes. Multiple studies have reported encouraging accuracy rates, with sensitivity and specificity often exceeding 90%. However, variability across studies highlights the need for contextual evaluation.

This study evaluates the diagnostic performance of Synovasure using culture-based results as the reference standard, offering a real-world analysis of its clinical utility where culture remains the primary diagnostic modality.

## Review

Methodology

Study Design and Patient Population

This retrospective study analysed 143 consecutive cases of clinically suspected PJI between April 2016 and January 2019 at a tertiary referral centre. Ethical approval was obtained, and all data were anonymised. Inclusion criteria were patients presenting with clinical suspicion of PJI, including joint pain, swelling, or reduced function, with or without systemic symptoms. Exclusion criteria included insufficient synovial fluid for testing or incomplete documentation of culture results.

Diagnostic Workflow

Patients underwent joint aspiration under sterile conditions. The skin was prepared with alcoholic chlorhexidine, and sterile drapes were applied. Fluid aspiration was performed using a 22-gauge spinal needle or Jamshidi needle. The skin was separately incised to avoid contamination. Image guidance was used for all aspirations, with fluoroscopy used for hips and ultrasound for knees. A dry tap was supplemented with intracapsular saline lavage to obtain a sample. Synovial fluid was divided for microbiological culture and Synovasure testing. For patients undergoing revision arthroplasty, intraoperative tissue samples were collected for culture with extended incubation protocols.

Definition of Infection

For the study, culture positivity in aspirate or intraoperative tissue was considered confirmatory of infection. Culture-negative samples were classified as non-infected. PJI treatment, however, was conducted based on the overall clinical context, incorporating intraoperative findings, serological and synovial fluid markers, and the treating surgeon’s judgement in accordance with established diagnostic criteria. This approach reflects the real-world complexity of PJI management, where culture results alone may not fully capture the presence of infection, particularly in cases of prior antibiotic exposure or low-virulence organisms. *Statistical Analysis*

Statistical analyses were conducted with SPSS version 20 (IBM Corp., Armonk, NY). Sensitivity, specificity, positive predictive value (PPV) and negative predictive value (NPV) were calculated with 95% confidence intervals (CIs).

Results

Cohort Characteristics

A total of 143 joints were evaluated, including 69 hips and 74 knees. The mean age was 65.5 years (standard deviation (SD) 13.9; range 29-93), with 54 males and 89 females. *Diagnostic Accuracy*

Of the 143 joints, 38 (26.6%) were culture-positive. Synovasure yielded 30 true positives, 29 false positives, 8 false negatives and 76 true negatives. *Performance Metrics*

**Figure 1:**
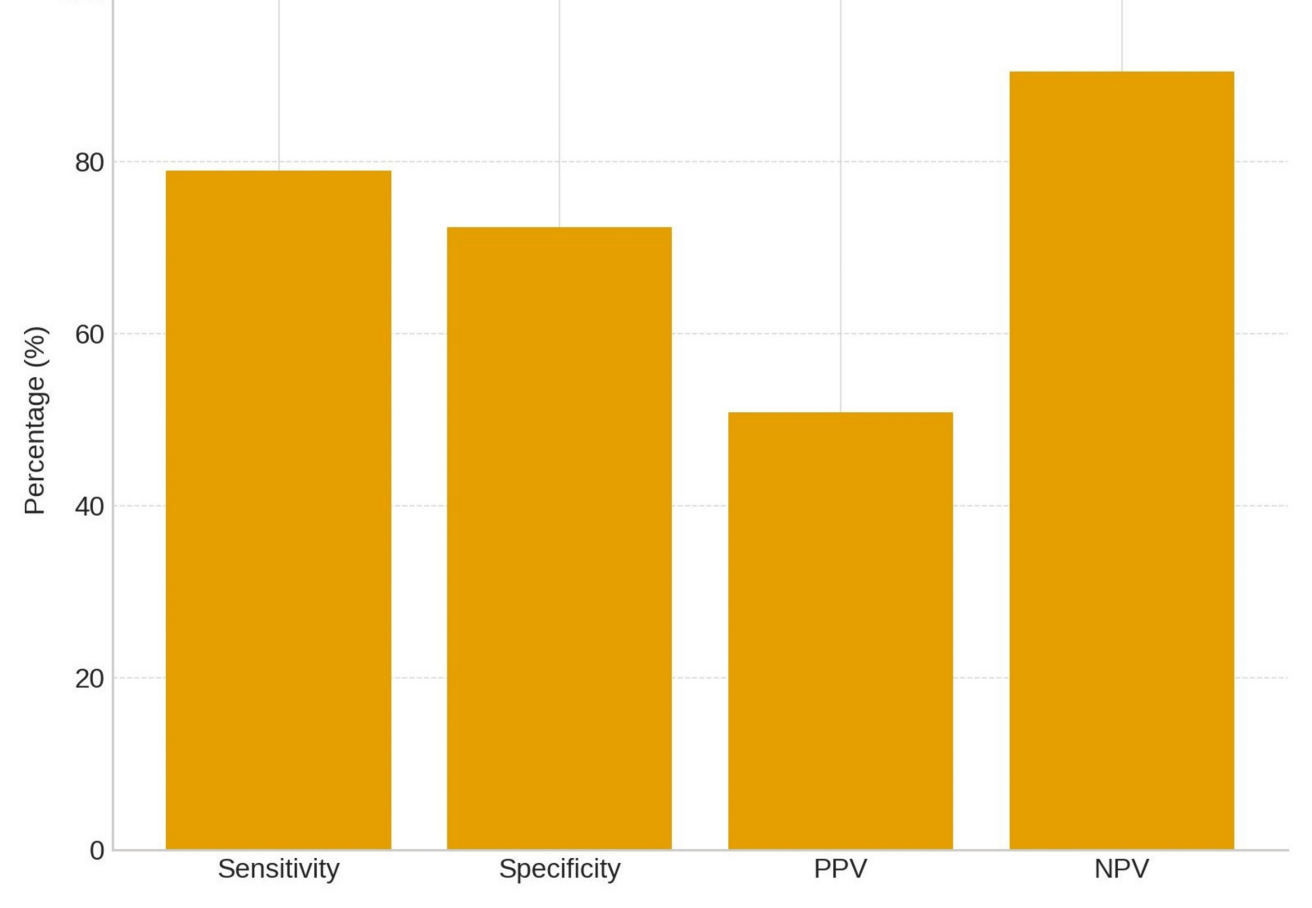
Diagnostic performance of the Synovasure alpha-defensin lateral flow test compared with culture-based results, demonstrating moderate sensitivity (78.95%) and specificity (72.38%) but a high negative predictive value (90.48%), highlighting its utility as a rule-out test for periprosthetic joint infection.

The sensitivity was 78.95% (95% CI: 62.68%-90.45%), specificity was 72.38% (95% CI: 62.80%-80.66%), PPV was 50.85% (95% CI: 42.15%-59.49%), and NPV was 90.48% (95% CI: 83.54%-94.68%), as reported in a study by Maximilian et al. (Figure 1) [7].

Discussion

This study demonstrates moderate sensitivity and specificity for the Synovasure alpha-defensin lateral flow (ADLF) test when compared with culture results. The high NPV underlines its potential as a rule-out test for periprosthetic joint infection (PJI), whereas the lower PPV suggests that it should not be used as a stand-alone diagnostic tool.

False-positive results were frequently observed in patients with a history of recent or ongoing antibiotic therapy, which may have suppressed bacterial growth in cultures while leaving inflammatory responses detectable by biomarker testing. In addition, underlying inflammatory or autoimmune conditions, such as rheumatoid arthritis or crystal arthropathy, contributed to elevated alpha-defensin levels in the absence of true infection.

Conversely, false negatives were commonly associated with low bacterial burden, particularly in early or indolent infections, or when organisms were slow-growing and difficult to detect [8]. These scenarios highlight the inherent diagnostic challenges and limitations of relying on a single modality.

Comparison With Literature

The diagnostic accuracy of the Synovasure alpha-defensin test has been evaluated in several studies, yielding variable results. For instance, Kuiper et al. demonstrated a sensitivity of 100% and specificity of 89%, employing the same Musculoskeletal Infection Society (MSIS) criteria [9]. In contrast, Ding et al. reported a sensitivity of 73.7% and specificity of 92.2%, using the MSIS criteria as the reference standard [10]. These discrepancies may arise from differences in study design, sample populations, and the handling of synovial fluid samples.

A meta-analysis by Zeng et al. pooled data from multiple studies and found that the lateral-flow version of the Synovasure test had a sensitivity of 77.4% and specificity of 91.3%, again using the MSIS criteria as the benchmark [2]. This lower sensitivity compared to laboratory-based tests underscores the potential limitations of the lateral-flow test in certain clinical settings.

**Figure 2:**
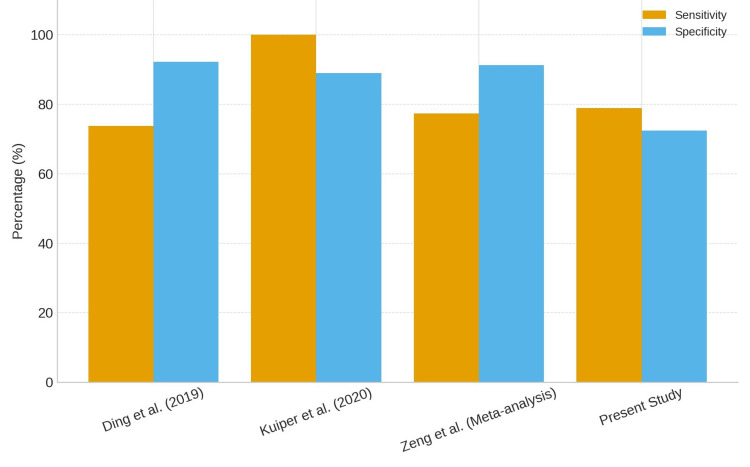
Comparison of diagnostic accuracy (sensitivity and specificity) of the Synovasure alpha-defensin test across published studies. The current study shows comparable sensitivity but lower specificity than previous reports, reflecting real-world variability and methodological differences such as benchmark criteria and sample handling.

Further studies have reported varying sensitivities and specificities for the Synovasure test, influenced by factors such as sample quality, timing of sample collection, and the presence of prior antibiotic treatment. These variables highlight the importance of standardising diagnostic protocols and considering the clinical context when interpreting test results [11,12]. The 2025 study further reinforces the findings of our research, highlighting that while the Synovasure ADLF test demonstrates a high NPV in the second stage of septic revision, its utility in the first stage is more limited, supporting the role of Synovasure as a rapid diagnostic tool for ruling out infection in later stages of PJI management (Figure 2) [13].

Clinical Implications

The rapid turnaround of Synovasure enhances its utility in clinical decision-making, particularly in excluding PJI at an early stage and thereby supporting more conservative or less invasive management pathways. Clinicians should be mindful of potential false positives in the context of inflammatory arthropathies and false negatives in cases of low bacterial load or slow-growing organisms [5,6].

Cost, Practicality and Comparative Utility

**Figure 3:**
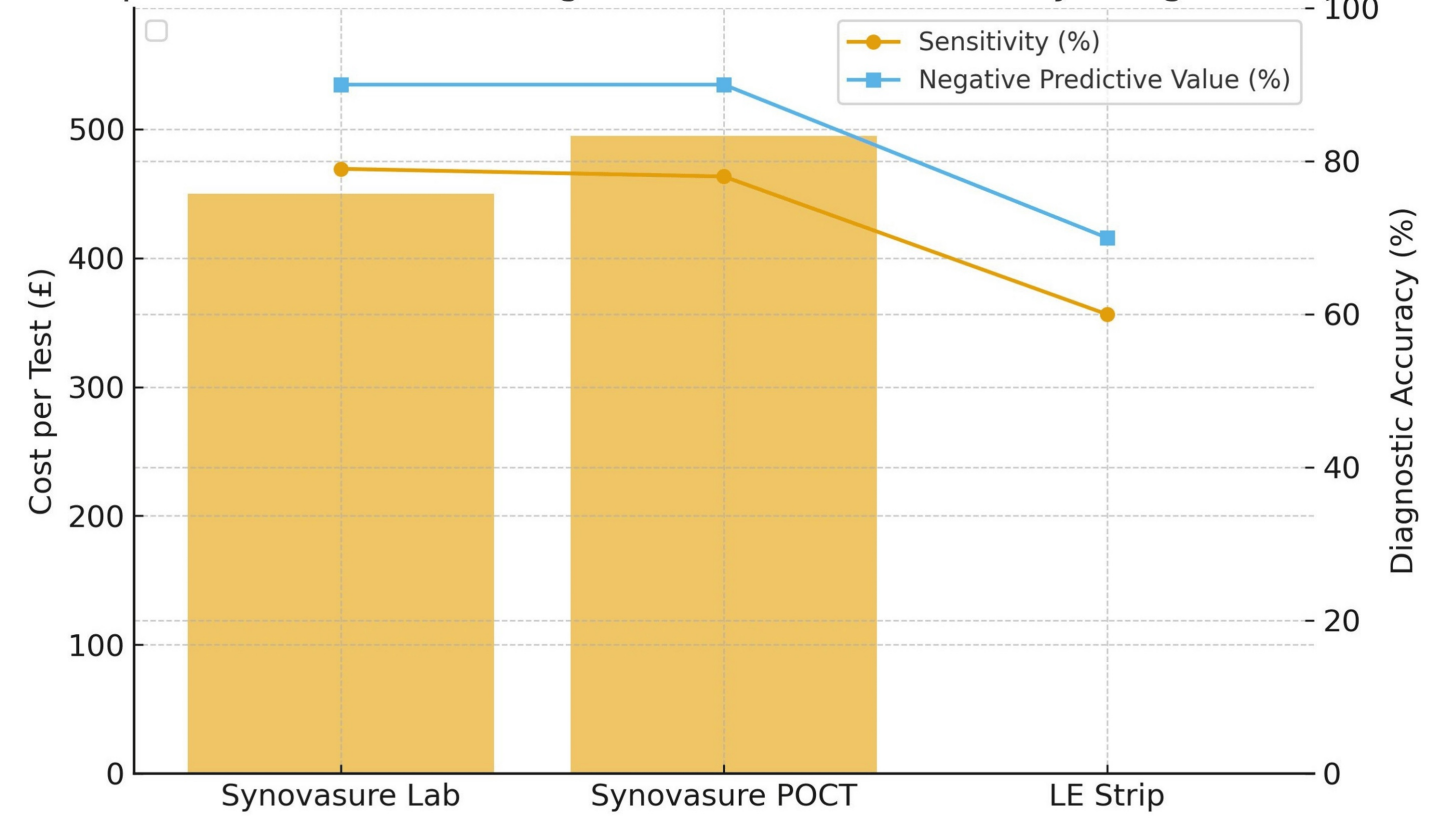
Comparative economic and diagnostic performance of Synovasure laboratory testing, Synovasure point-of-care testing (POCT), and the leukocyte esterase (LE) strip. Synovasure Lab and POCT demonstrate substantially higher per-test costs (£450 and £495, respectively) compared with the near-zero cost of the LE strip (£0.11). Despite this disparity, both Synovasure modalities exhibit moderately higher sensitivity (approximately 78%-79%) and superior negative predictive value (NPV ≈ 90%) relative to the LE strip. These findings highlight the trade-off between diagnostic performance and economic burden, underscoring the importance of context-specific test selection in prosthetic joint infection (PJI) workup.

The Synovasure alpha-defensin test presents a substantial cost compared to traditional diagnostic methods. In the United Kingdom, the laboratory-based Synovasure test is priced at approximately £450 per sample, while the point-of-care lateral-flow version is priced around £495 each, or £300 when purchased in bulk. In contrast, the leucocyte esterase (LE) strip test is significantly more economical, with costs ranging from £0.11 to £0.40 per strip, depending on the supplier and quantity purchased (Figure 3) [2].

Health Technology Wales (HTW) conducted an economic evaluation of Synovasure for diagnosing PJI. The report was published in 2018. The analysis indicated that the laboratory-based Synovasure test, when used as part of a diagnostic bundle, resulted in an incremental cost-effectiveness ratio (ICER) exceeding £20,000 per quality-adjusted life year (QALY), which is above the commonly accepted cost-effectiveness threshold in the United Kingdom. Conversely, the point-of-care lateral-flow version demonstrated a more favourable ICER of approximately £19,000 per QALY for knee revisions, suggesting potential cost-effectiveness in this specific clinical scenario.

Practically, LE strips are advantageous for initial screening due to their low cost and immediate results. However, their diagnostic reliability is limited by factors such as operator subjectivity and susceptibility to interference from blood or other substances. Synovasure offers enhanced diagnostic accuracy, particularly in equivocal cases, with a high NPV. Nonetheless, its higher cost and the need for aspiration procedures may limit its routine use in settings with constrained resources.

Challenges in Diagnosing PJI and Implications for Patients

PJI remains a notoriously difficult condition to diagnose, as no single test offers perfect accuracy. Factors such as prior antibiotic use, low-grade infections, and the presence of inflammatory arthropathies can confound both conventional and novel diagnostic tools [5,6,8]. The consequences of diagnostic uncertainty are significant: underdiagnosis risks persistent infection, implant failure, and the need for revision surgery, while overdiagnosis can lead to unnecessary operations, prolonged antibiotic therapy, and avoidable patient morbidity. In this context, Synovasure may provide reassurance when ruling out infection, but its role in confirming PJI is less robust, necessitating integration with a broader diagnostic framework.

Limitations

This study has several limitations. First, its retrospective design introduces inherent bias and limits control over confounding factors. Second, there may be significant inter-practitioner variability in aspiration technique, potentially affecting sample quality. Third, we compared Synovasure results only against microbiological culture, without incorporating histological or molecular methods, and therefore may have under-represented culture-negative infections. Finally, the study was conducted at a single centre, which may limit generalisability. Prospective multicentre studies employing standardised criteria and incorporating cost-effectiveness analyses are warranted to better define the role of Synovasure within the diagnostic pathway for PJI.

## Conclusions

Synovasure alpha-defensin testing provides rapid results with excellent NPV, making it a useful adjunct for ruling out periprosthetic joint infection. However, its moderate sensitivity, specificity, and low PPV in this real-world cohort highlight that it cannot reliably confirm infection when used alone. False positives in inflammatory conditions and false negatives in low-grade infections reinforce the need for multimodal evaluation. While Synovasure may enhance diagnostic confidence when integrated into established criteria, its cost and variable performance warrant cautious, selective clinical use. Larger prospective studies are needed to clarify its definitive role in PJI diagnosis.
